# Grade III meningioma with gastro-intestinal tract and brain metastases: case report and review of the literature

**DOI:** 10.1186/s12957-019-1596-6

**Published:** 2019-04-16

**Authors:** Laura Bender, Benoit Lhermitte, Hélène Carinato, Seyyid Baloglu, Mehdi Helali, Hélène Cebula, Delphine Antoni, Georges Noel

**Affiliations:** 1 Radiotherapy Department, Centre Paul Strauss, UNICANCER, 3, rue de la Porte de l’hôpital, F-67065 Strasbourg, France; 2 Pathology Service, University Hospital Hautepierre, 1, rue Molière, 67000 Strasbourg, France; 3 Medical Oncology Department, Centre Paul Strauss, UNICANCER, 3, rue de la Porte de l’hôpital, F-67065 Strasbourg, France; 4 Radiology Department, University Hospital Hautepierre, 1, rue Molière, 67000 Strasbourg, France; 5 Nuclear Medecine Department, Centre Paul Strauss, UNICANCER, 3, rue de la Porte de l’hôpital, F-67065 Strasbourg, France; 6 Neurosurgery Department, University Hospital Hautepierre, 1, rue Molière, 67000 Strasbourg, France; 7 CNRS, IPHC UMR 7178, Centre Paul Strauss, UNICANCER, Université de Strasbourg, 67000 Strasbourg, France

**Keywords:** Bevacizumab, Brain metastases, Gastro-intestinal tract metastases, Malignant meningioma, Systemic treatment

## Abstract

**Background:**

Meningioma is the most common adult primary intracranial tumor. Malignant meningioma is a rare variant of meningioma. The prognosis for the patients with these tumors is poor, due to the tumor’s capacity for relapse and to develop distant metastases. These tumors can present the same evolutionary course as aggressive carcinoma.

**Case description:**

We report the case of distant brain and gastro-intestinal tract (GIT) metastases. A 78-year-old patient developed malignant meningioma with a Ki-67 proliferative index of 40%. According to guidelines, surgery followed by postoperative radiotherapy (RT) was performed. Three months after the end of RT, he presented histologically proven meningioma distant brain and GIT metastases.

**Conclusions:**

To our knowledge, this is the first case of meningioma GIT metastases. Also, we report the difficulty to confirm the diagnosis of meningioma metastases. Indeed, malignant meningioma has the same histopathological features as melanoma or carcinoma. The standard of care for the management of malignant meningioma is gross total surgery followed by postoperative radiotherapy. Metastatic meningioma is uncommon and no guidelines for the management of recurrent or metastatic meningioma have yet been published. However, several studies reported systemic therapeutic options such as antibody against VEGF, somatostatin analogs, PDGF-R, and VEGF-R tyrosine kinase inhibitors, in the case of recurrent or metastatic meningioma. We also made a review of the actual literature of systemic treatment options for metastatic meningioma.

## Background

Meningiomas are the most common adult primary central nervous system tumors. The Central Brain Tumor Registry of the United States (CBTRUS) reported 129,841 new cases between 2008 and 2012. In the USA, meningiomas represent 36.4% of all cases of primary central nervous system tumors [1, 2]. Meningiomas derive from arachnoid cap cells located in arachnoid villi. These tumors arise in the majority of the cases from brain meninges but 10% derive from spinal cord meninges [3]. Immunohistochemical analysis reveals an expression of vimentin, protein S100, epithelial membrane antigen, and progesterone receptors [4, 5]. Ragel et al. described aberrant signaling pathways (mammalian Target of Rapamycin (mTOR), Phosphoinositide 3-kinase (PI3K), Mitogen Activated Protein Kinase (MAPK)) implicated in meningioma tumorigenesis [6]. Pavelin et al. confirmed a statistically significant correlation between Ki-67 rate and the WHO classification. Indeed, the median Ki-67 rate was 1.5% (range 0–13.9) for benign meningioma compared to 10.2% (3.4–42.1) for anaplastic meningioma [7]. According to the World Health Organization (WHO) 2016 classification, meningiomas are divided into three grades: grade I or benign meningioma, grade II or atypical meningioma, and grade III or malignant meningioma. The WHO 2016 classification does not undergo revisions about the classification and the grading of meningioma compared to the WHO 2007 classification. The only change is that brain invasion is a criterion, which suffice for diagnosing grade II meningioma. Grade I meningiomas (nine subtypes) represent the most common variant. These tumors have a good prognosis with 10-year progression-free survival (PFS) rate from 75 to 95% and 10-year overall survival (OS) rate from 80 to 90% [8]. Grade II meningiomas (atypical, clear-cell, and chordoïd) have a poor prognosis with 10-year PFS rate from 23 to 78% and 10-year OS rate from 50 to 79% [8]. Grade III meningiomas (anaplastic, papillary, and rhabdoïd) are a rare variant of meningioma. These tumors represent 1.2% of all meningioma. The Central Brain Tumor Registry of the United States counted 3004 new cases of malignant meningioma between 2000 and 2010 [3]. The age-adjusted incidence rate is equally distributed between genders (0.08/100,000 female population and 0.09/100,000 male population) [3]. Grade III meningiomas are defined by 20 or more mitoses per ten high power fields and/or pathological examinations, which look like pseudo-carcinomas, pseudo-melanomas, or high grade pseudo-sarcomas [9]. These tumors have a worse prognosis with 10-year PFS rate of 0% and 10-year OS rate from 14 to 34% [8]. Moreover, malignant meningioma may develop distant metastases. Enam et al. reported a metastases incidence of 43% for grade III meningioma compared to 0.76% when considering all meningiomas [10]. The lung (37.2%), bone (16.5%), intraspinal (15.2%), and liver (9.2%) were the most frequent metastases localizations [10]. There are actually no recommendations concerning the management of distant metastases of meningioma. Several systemic treatments such as antibody against vascular endothelial growth factor (VEGF), tyrosine kinase inhibitors, and somatostatin analogs were studied in cases of metastatic or recurrent meningioma. We proposed a review of actual literature in the paragraph discussion.

## Case presentation

**Fig. 1:**
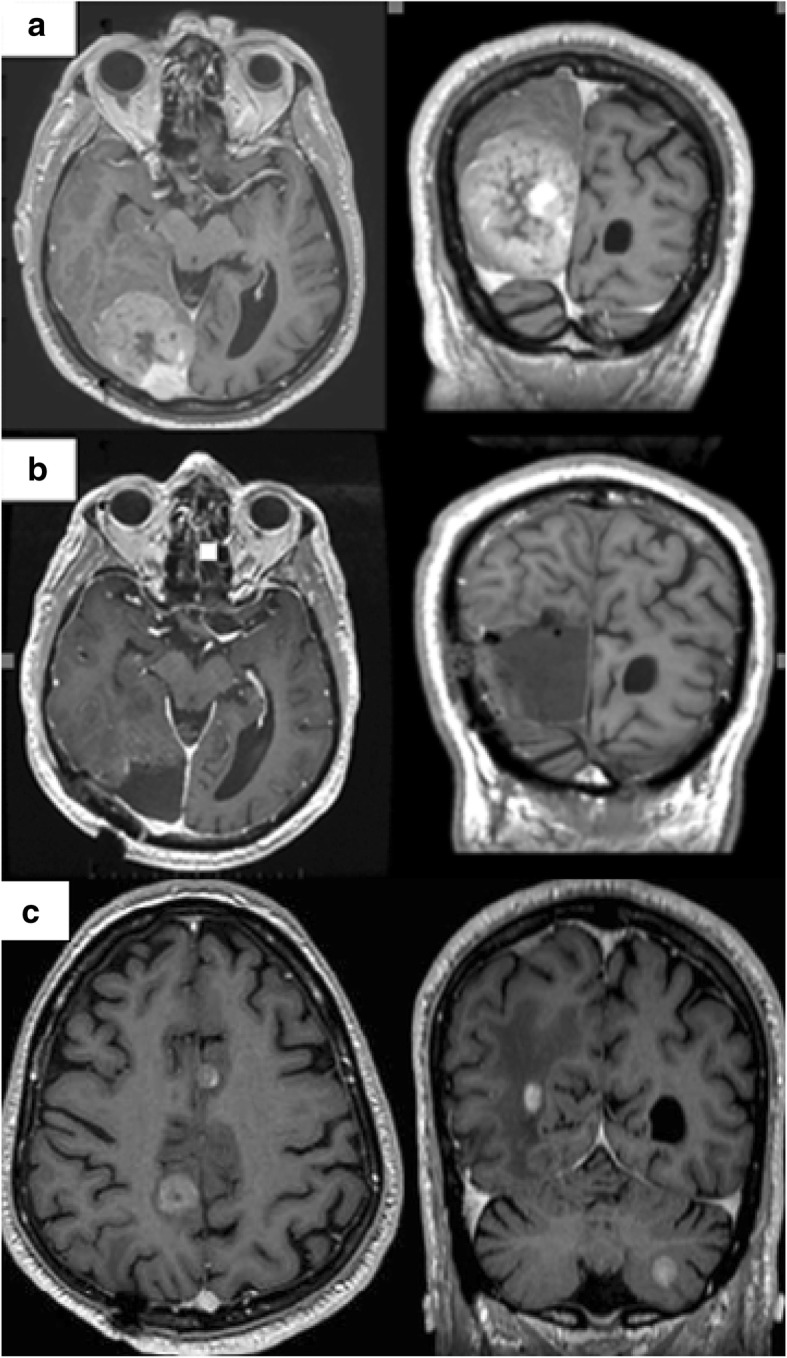
MRI-enhanced axial and coronal T1-weighted imagings. **a** An extra-axial lesion in the right occipital lobe with a mass effect of the posterior horn of the right ventricle. **b** Gross total resection of the right occipital meningioma. **c** Distant brain metastases of malignant meningioma

**Fig. 2:**
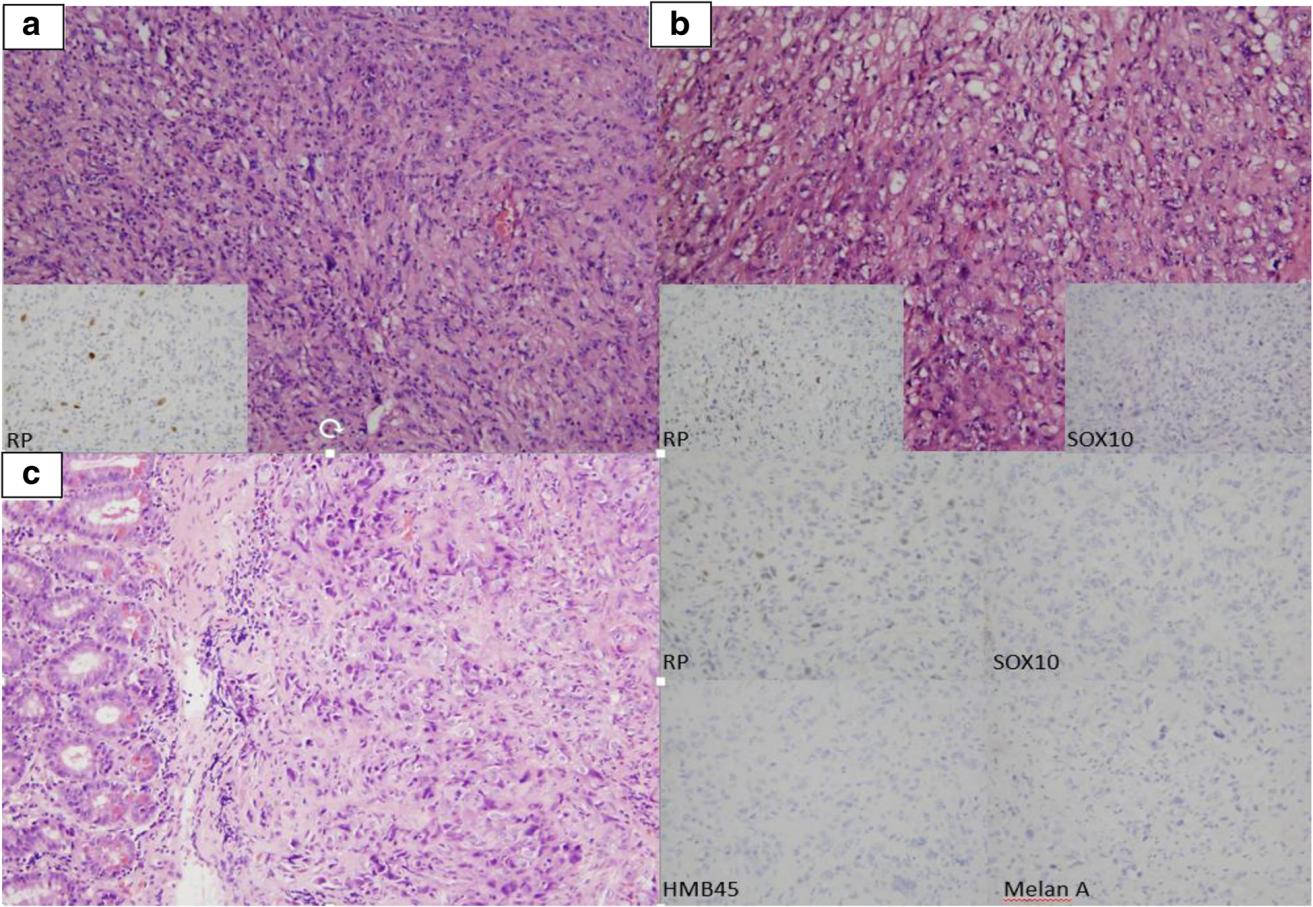
Histological features of intracranial and intestinal samples. **a** Resection of the right occipital lesion: pseudosarcomateous proliferation of atypical fusiform cells with focal progesterone receptors and EMA (not shown) expression consistent with a malignant meningioma, WHO grade III. **b** Resection of one of the new occipital lesion showing the same proliferation with focal expression of progesterone receptors. Melanoma’s markers, such as SOX10 or HMB45 and Melan A (not shown) were not expressed. **c** Intestinal resection showing a morphologically similar proliferation involving the mucosa and the sub-mucosa. Immunohistochemical phenotype was similar too with focal expression of progesterone receptors and negativity of melanocytic markers (SOX10, HMB45, and Melan A).

**Fig. 3:**
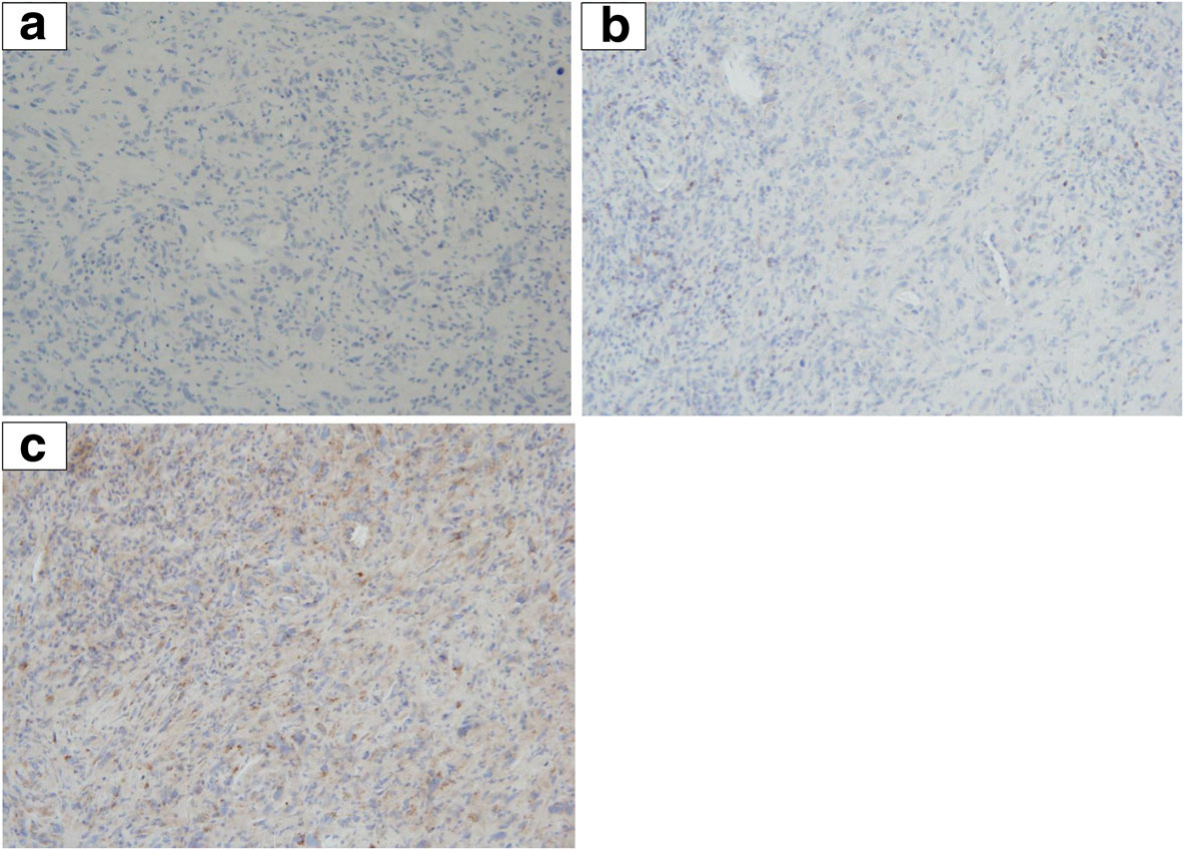
Histological features of intracranial and intestinal samples. **a** Resection of the right occipital lesion: pseudosarcomateous proliferation of atypical fusiform cells with focal progesterone receptors and EMA (not shown) expression consistent with a malignant meningioma, WHO grade III. **b** Resection of one of the new occipital lesion showing the same proliferation with focal expression of progesterone receptors. Melanoma’s markers, such as SOX10 or HMB45 and Melan A (not shown), were not expressed. **c** Intestinal resection showing a morphologically similar proliferation involving the mucosa and the sub-mucosa. Immunohistochemical phenotype was similar too with focal expression of progesterone receptors and negativity of melanocytic markers (SOX10, HMB45, and Melan A)

**Fig. 4:**
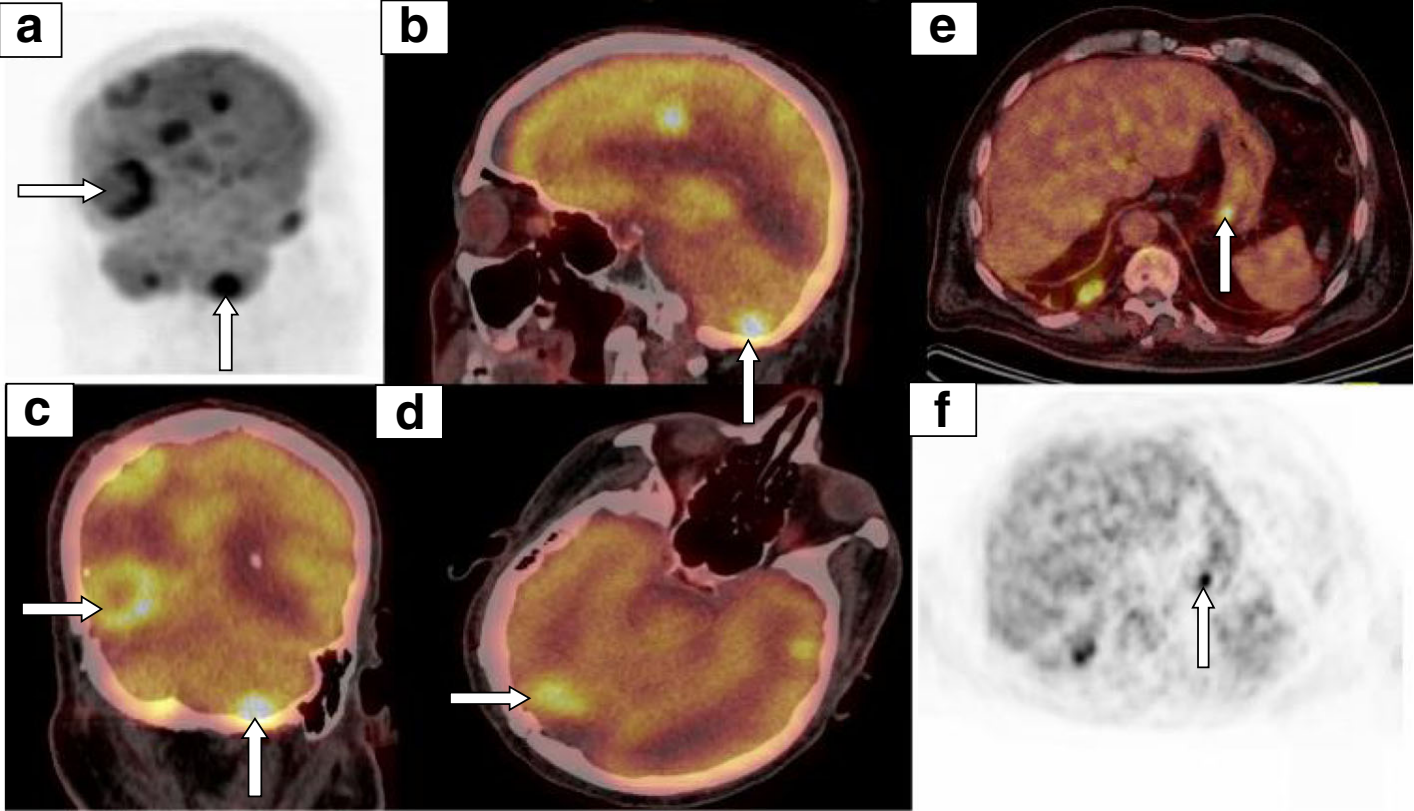
^18^FDG TEP TDM. **a**, **b**, **c**, **d** Hypermetabolic cerebral and cerebellar lesions related to secondary lesion of malignant meningioma. **e**, **f** Hypermetabolic lesion at the fundus

A 78-year-old man with a medical history of hypertension, hypercholesterolemia, aneurysm of the ascending aorta, and chronic inflammatory pleurisy presented headaches and visual disturbances (left homonymous hemianopia). Brain magnetic resonance imaging (MRI) revealed an occipital extra-axial lesion with surrounding edema (Fig. 1a). Three weeks later, the patient underwent a total resection, which revealed a malignant meningioma with Ki-67 proliferative index of 40% (Fig. 2a). Next-generation sequencing (NGS) detected no specific mutation. Immunohistochemical analysis found high expression of pankeratin AE1/AE3, vimentin, INI-1 (clone MRQ-27), and focal expression of epithelial membrane antigen. P53, cytokeratin 7, and cytokeratin 20 were negative. All melanocytic makers (HBM45, SOX10, Melan A) were negative. Moreover, there was no expression of STAT-6 (Fig. 3a), bcl-2 (Fig. 3b), and a nonspecific granular cytoplasmic staining of CD99 (Fig. 3c). Postoperative brain MRI showed hemorrhagic remodeling without any evidence of a residual tumor (Fig. 1b). According to the actual data of the literature, postoperative surgical bed irradiation with total dose of 68 Gy (34 daily fractions of 2 Gy) was performed. At the end of RT, the patient was in a good health condition without neurologic symptoms. One week after the end of RT, he underwent a total resection of a right shoulder cutaneous lesion. Histopathological analysis revealed a superficial spreading melanoma. Four months after the end of RT, the patient presented dizziness and left arm weakness. A brain MRI revealed a local recurrence and six new brain lesions (Fig. 1c). In order to distinguish melanoma brain metastases between meningioma brain metastases, the occipital lesion was biopsied. Pathological analysis confirmed WHO grade III meningioma with Ki-67 proliferative index (MIB-1) of 80%. Immunohistochemical analysis revealed a focal expression of progesterone receptor (Fig. 2b) without any expression of melanocytic markers (SOX10, HMB45, Melan A). Thus, a hypothesis of melanoma brain metastases was excluded. Positron emission tomography with radiolabeled [18F]-fluoro-2-deoxy-D-glucose coupled to a CT-scan (^18^FDG PET/CT) showed six hypermetabolic cerebral and cerebellar lesions (Fig. 4a, b, c, d), a focal intense uptake lesion of the fundus (Fig. 4e, f), and a sigmoidal nodule. The results of the brain MRI, ^18^FDG PET/CT, and the pathological examination of the brain lesion suggested that the patients developed several distant brain metastases of a malignant meningioma. No specific treatment was initiated. Five weeks after the cerebral biopsy, the patient presented a digestive hemorrhage. Gastroscopy showed many duodenal micro-ulcerations; no biopsy was made. He had a second digestive hemorrhage 5 days later, which required hemostatic surgery. Pathological examination of a gastro-intestinal tract specimen revealed a malignant lesion, which had the same morphological and immunohistochemical features of the right occipital lesion (Fig. 2c). Again, there was also no specific mutation on NGS. Radiation therapy of the whole brain was performed (30 Gy in 10 fractions of 3 Gy). One month after the end of RT, despite the lack of guidelines, a systemic treatment with bevacizumab (10 mg/kg intravenous every 2 weeks) was administrated. Only one injection was made. The patient died suddenly on the 15th of June. The main hypothesis of the cause of death was a cardiac arrest secondary to a pulmonary embolism. An autopsy was not proposed to the family to understand the cause of death that is why the post-mortem examination was not performed to determine the real cause of death.

## Discussion and conclusions

We reported a rare case of meningioma cerebral and gastro-intestinal tract metastases. To our knowledge, this is the first case of histological proven meningioma duodenal metastases. Indeed, Surov et al. described 164 distant metastases of meningioma. Their review reported no digestive localization [11]. Moreover, our presentation highlighted the difficulty to confirm the diagnosis of meningioma metastases, especially in our case since the patient presented a synchronous melanoma. Mawrin et al. showed that malignant meningioma and melanoma presented similar morphological features. Immunohistochemistry was necessary to distinguish between melanoma metastases and meningioma metastases [12]. Furthermore, the second differential diagnosis of malignant meningioma is hemangiopericytoma. Indeed, both tumors present similar histological and immunohistochemical features [13]. In order to distinguish between these tumors, we performed additional immuohistochemical analysis like STAT-6, bcl-2 and CD99. Meningeal hemangiopericytoma can develop distant extraneuronal metastasis with a 5-year metastasis rate between 4% and 20% and a 10-year metastasis rate between 25% and 34% [14–16]. The most common extraneuronal sites are the bone, lung, and liver [14]. Takahashi et al. described a case of gastro-intestinal stromal tumor (GIST) with hemangiopericytoma-like histological features. This case suggested that additional immunohistochemical and mutations analysis of the KIT gene should be performed in case of primary brain lesion with distant gastro-intestinal tract lesions; in order to distinguish between meningioma gastro-intestinal tract (GIT) metastases, hemangiopericytoma GIT metastases, and GIST with hemangiopericytoma-like histological pattern [17].

**Fig. 5:**
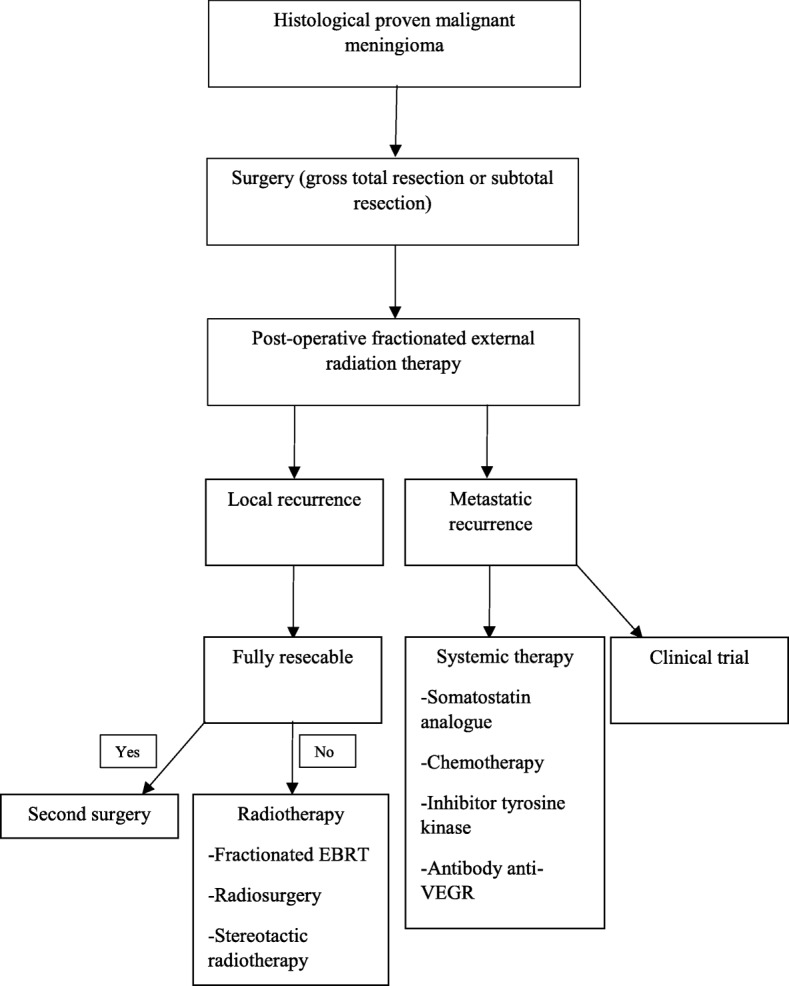
Management strategy of grade III meningioma

Furthermore, management of malignant meningioma is based on surgery followed by fractionated external beam radiation therapy [18, 19]. However, actually no guidelines for management of recurrent or metastatic grade III meningioma are published [18]. Furthermore, we made a review of the actual literature of systemic therapy options for the management of malignant meningioma and we proposed a management algorithm (Fig. 5).

Several studies suggested systemic therapies like chemotherapy, somatostatin analogs, tyrosine kinase inhibitors, and anti-angiogenic in the cases of recurrent or metastatic meningioma.

Somatostatin is produced in the hypothalamus and inhibits tumor cell proliferation. Five subtypes of somatostatin receptors (from sstr1 to sstr5) have been characterized [20]. Meningiomas have a high expression of somatostatin receptors (90%) especially the sstr2 subtype [20]. Simo et al. included five grade II and four grade III recurrent meningiomas in a phase II prospective trial. Indium-111 octreotide single-positron emission computed tomography (SPECT) was positive in all cases which confirmed the sstr2 expression. Brain MRI was performed every 3 months. Patients underwent a median of three cycles of treatment (range 1–8). No complete or partial response was observed. Disease stability rate was obtained in 33% of patients. Median overall survival time was 18.7 months (range 2.7–39.9) [20] (Table 1).

**Table 1:** 6-month Progression-free survival and median overall survival for recurrent or metastatic malignant meningioma treated with systemic therapy

Authors	Year	Study type	Drug used	Grade III meningioma (#)	6-month PFS (%)	Median OS (month)
Mazza et al. [25]	2016	Phase II, prospective	Hydroxyurea	8	NA	27.5
Mazza et al. [25]	2016	Phase II, prospective	Hydroxyurea + imatinib	7	NA	6
Kaley et al. [26]	2015	Phase II, prospective	Sunitinib	6	44	24.6
Simo et al. [20]	2014	Phase II, prospective	Octreotide	4	44	18.7
Raizer et al. [23]	2014	Phase II, prospective	Valatanib	8	37.5	23
Lou et al. [21]	2012	Retrospective	Bevacizumab	3	87.5	NA

*NA* Not Available

According to Ragel et al. [6], meningiomas have a vascular endothelial growth factor (VEGF) and express VEGF-receptor (VEGF-R) in a rate of 84% and 67%, respectively. VEGF-R activation leads to tumor angiogenesis and cerebral edema [6]. Lou et al. studied 11 grade II and 3 grade III recurrent meningiomas who were treated with bevacizumab, a human antibody against VEGF. Six-month PFS rate was 87.5% [21] (Table 1). Furtner et al. showed a cerebral edema reduction for patients that were treated with bevacizumab [22]. Raizer et al. included in a prospective study 17 grade II and 8 grade III recurrent or metastatic meningiomas. All patients were treated with valatanib (anti VEGF-R). Considering malignant meningioma, 6-month PFS rate was 37.5%. Median PFS and OS times were 3.6 months and 23 months, respectively. The authors described only one case of grade 4 toxicity (transaminase elevation) [23] (Table 1).

Meningiomas have platelet-derived growth factor receptors (PDGF-R) with a rate of 80%. Platelet-derived growth factor (PDGF) is a growth factor, which stimulates MAPK pathway and PI3K pathway [24]. A phase II trial studied 15 recurrent or metastatic grades I, II, and III meningiomas. Seven patients were included in arm A with hydroxyurea (HU) and imatinib (anti PDGF-R). Eight patients were included in arm B with HU alone. Nine-month PFS rate was higher in arm B alone compared to arm A (75% and 0%, respectively, *p* value not shown). However, there were not enough patients included and the study was prematurely stopped [25] (Table 1).

Sunitinib is a PDGF-R and VEGF-R tyrosine kinase inhibitor. Kaley et al. described a 6-month PFS rate of 44% for recurrent grade II and grade III meningioma treated with sunitinib. Median OS rate was 24.6 months. Moreover, median PFS time for patients with expression of VEGF-R was 6.4 months compared to 1.4 months (*p* = 0.05) for patients without an expression of VEGF-R [26] (Table 1).

Several trials, which studied systemic therapy for recurrent and metastatic meningioma, were performed. However, not enough patients were included to determine the most efficient drug.

## Conclusion

Malignant meningioma is a rare disease with a poor prognosis. These tumors have a high ability to relapse and to metastasize. Surgery followed by radiation therapy is recommended for the management of grade III meningioma. However, no consensus for the management of recurrent or metastatic meningioma was established. Bevacizumab, a human antibody against VEGF-R, seemed to be partially effective. Further prospective, multicenter studies are necessary to confirm these outcomes. Eventually, the discovery of specific molecular markers or driver mutations will permit us to propose a personalized treatment. Actually, due to the poor prognosis of recurrent or metastatic meningioma, patients should be included in prospective trials.

## Abbreviations

^18^FDG PET/CT: Positron emission tomography with radiolabeled [18F]-fluoro-2-deoxy-d-glucose coupled to a CT-scan
CBTRUS: Central Brain Tumor Registry of the United States
GIST: Gastro-intestinal stromal tumor
GIT: Gastro-intestinal tract
HU: Hydroxyurea
MAPK: Mitogene Activated Protein kinase
MRI: Magnetic resonance imaging
mTOR: Mammalian Target of Rapamycin
NA: Not applicable
NGS: Next-generation sequencing
OS: Overall survival
PDGF: Platelet-derived growth factor
PDGF-R: Platelet-derived growth factor receptors
PFS: Progression-free survival
PI3K: Phosphoinositide 3-kinase
RT: Radiotherapy
SPECT: Single-positron emission computed tomography
VEGF: Vascular endothelial growth factor
VEGF-R: Vascular endothelial growth factor receptors
WHO: World Health Organization
